# Brain structural correlates of postoperative axial pain in degenerative cervical myelopathy patients following posterior cervical decompression surgery: a voxel-based morphometry study

**DOI:** 10.1186/s12880-023-01057-8

**Published:** 2023-09-19

**Authors:** Aixian Tian, Hongzhi Gao, Zhan Wang, Na Li, Jianxiong Ma, Lin Guo, Xinlong Ma

**Affiliations:** 1 Orthopedic Research Institute, Tianjin Hospital, Tianjin University, Jiefang Nan Road 406, Hexi District, Tianjin, 300211 P. R. China; 2 Radiology Department, Tianjin Hospital, Tianjin University, Jiefang Nan Road 406, Hexi District, Tianjin, 300211 P. R. China

**Keywords:** Degenerative cervical myelopathy, Central sensitization, Postoperative axial pain, Support vector machine, Voxel-based morphometry, fMRI

## Abstract

**Objective:**

To investigate the brain structural correlates of postoperative axial pain (PAP) in degenerative cervical myelopathy (DCM) following posterior cervical decompression surgery.

**Methods:**

Structural images with high-resolution T1 weighting were collected from 62 patients with DCM and analyzed, in addition to 42 age/gender matched subjects who were healthy. Voxel-based morphometry (VBM) was analyzed, grey matter volume (GMV) was computed. One-way ANOVA was performed to reveal the GMV differences among DCM patients with PAP, patients without PAP and healthy controls (HC). Post-hoc analyses were conducted to identify the pair-wise GMV differences among these three groups. Analyses of correlations were conducted to uncover the link between clinical measurements and GMV variations. Last, support vector machine (SVM) was conducted to test the utility of GMV for classifying PAP and nPAP DCM patients.

**Results:**

Three main findings were observed: [1] Compared to healthy controls, DCM patients showed a significantly lower GMV in the precuneus preoperatively. DCM patients with PAP also exhibited a lower GMV within precuneus than those without; [2] In DCM patients with PAP, the precuneus GMV was inversely related to the postoperative pain intensity; [3] Moreover, successful classification between PAP and nPAP were observed via SVM based on precuneus GMV as features.

**Conclusion:**

In summary, our results indicate that precuneus GMV may be linked to PAP in DCM, and could be employed to forecast the emergence of PAP in DCM patients.

## Introduction

Degenerative cervical myelopathy (DCM), marked by degenerative alterations in the cervical spine, is commonly seen in clinical practice and requires decompression of the spinal canal to avert further neurological damage [1]. To date, surgery has been considered as the primary treatment for DCM patients and a surgical procedure at the start of myelopathy may significantly improve the chances of recovery [2, 3]. Despite the controversy surrounding surgical strategy for DCM over the past decade (e.g., posterior vs. anterior), posterior laminectomy and laminoplasty are still the common treatment options for multiple-level lesions [4]. Clinical results are effective and surgical-related side effects are lower when compared to anterior approaches [5, 6]. Nevertheless, postoperative axial pain in the area from the nuchal to the periscapular region, which significantly interfere with the quality of life of DCM patients postoperatively, has been largely ignored and its associated factors have still been controversial [7]. In the current state of perioperative management, there is no effective method for preventing or reducing this vexing complication [8]. A systematic investigation of its potential mechanisms is therefore warranted. Recently possible assumption has been made by Zheng et al. The researchers studied the pain thresholds, adding of pain over time, and the modulation of pain in DCM patients and found that there is a possibility that axial pain might be aggravated by endogenous pain modulation deficit prior to posterior decompression surgery in DCM patients [9]. Their results indicated that preoperative pain hypersensitivity might be a contributing factor to the presence of PAP following surgery. Despite this, the neural correlates behind such phenomenon remain mysterious and identifying the neural correlates of PAP could be beneficial in order to classify DCM patients during the period prior to surgery and create new strategies for pain alleviation in DCM patients [8].

In the past decades, researchers have identified various brain regions, which are generally activated during nociceptive stimuli, to be participated in pain perception including the primary (S1) and secondary (S2) somatosensory cortex, the thalamus, the anterior cingulate cortex (ACC), and the insula [10–13]. Aside from the pain matrix, some anticorrelated deactivated areas, particularly the default mode network (DMN) that consists of precuneus, medial frontal cortex and posterior cingulate cortex (PCC) have been shown to be related to pain perception but have been relatively overlooked [14, 15]. In many chronic pain conditions, abnormal grey matter volume within DMN and pain matrix have been identified [15–19]. In these studies, voxel-based morphometry, which is a widely used metric for measuring brain morphometry changes, has received much attention due to its interpretability, simplicity and replicability. Recent studies have shown that the grey matter density within precuneus was significantly correlated with the inter-individual differences in pain sensitivity [15]. These results provided new insights for investigating neural correlates of PAP in patients with DCM underwent posterior decompression surgery.

In our research project, we employed voxel-based morphometry analysis along with several psychological questionnaires assessing pain-related traits to examine the link between preoperative pain sensitivity and PAP in patients existing DCM, as well as its relationship to grey matter changes; and to assess if brain morphometry can be used to accurately predict whether PAP will happen in DCM patients.

## Method

### Subjects

This research project was reviewed and approved by the respective institutional review board of Tianjin Hospital, Tianjin, China, and all participants provided written consent prior to taking part in any of the activities. We confirmed that all methods were performed in accordance with the relevant guidelines and regulations. Sixty-two DCM patients who fit the following criteria were included in this study: [1] cervical myelopathy identified by MRI along the cervical spine; [2] clinical signs and symptoms corresponding to myelopathy identified by MR; [3] no prior history of other spine diseases or surgery, and willing to accept posterior decompression surgery (e.g., laminoplasty, laminectomy); [4] able to complete MR scan; [5] no narrowing of the carotid artery or the extracranial vertebral artery as determined by Doppler ultrasound; [6] no signs of any other neurological, ocular, psychiatric, or systemic illnesses such as diabetes and hypertension; and [7] no prior use of alcohol or drug misuse.

Forty-one individuals of healthy physical and mental status were recruited via posters and provided written authorization before participation and make true the participants with [1] no signs of spinal cord compression; [2] no evidence of other spinal, brain, or systemic disorders; [3] capable of completing fMRI scans; [4] no signs of any other neurological, ocular, psychiatric, or systemic illnesses such as diabetes and hypertension; and [5] no prior use of alcohol or drug misuse, no fear of anxiety, no fear of confined spaces, and no ferromagnetic implants. Thus, a total of 62 DCM individuals and 41 healthy participants were contained in the research.

### Questionnaires

Each participant first underwent a high-resolution anatomical MRI, and then proceeded to another room to complete the questionnaires in the validated Chinese version. In line with previous studies [10], four questionnaires (Pain Anxiety Symptoms Scale [20], Fear of Pain Questionnaire III [21], Pain Catastrophizing Scale [22] and Pain Vigilance and Awareness Questionnaire [23]) were applied to measure fear of pain (FoP). All the scores of the questionnaires were z-normalized across participants, and an average was taken to calculate a single measure of FoP afterwards.

### Postoperative axial pain (PAP) vs. non-PAP

The Japanese Orthopaedic Association (JOA) score, a widely used tool for assessing the severity of DCM, was administered to all DCM patients. A numerical rating scale (NRS) ranging from 0 to 10 (1 being no pain, 2 representing threshold pain, 10 representing unbearable pain) was used to measure preoperative neck pain when they first admitted to hospital. We requested the patients to quantify the mean strength of neck pain in the prior month. NRS was used to measure postoperative neck pain intensity once at the one-year follow-up by telephone. The participants were asked to report the mean strength of axial neck pain they had experienced in the past month. DCM patients were classified as postoperative axial pain (PAP) and non-postoperative axial pain (nPAP) groups, based on the postoperative NRS rating, with a cut-off value of 4 or more, in accordance with previous studies.

### Image acquisition

The T1 images were collected within 1 week in DCM patients before decompression surgery. Structural T1-weighted high-resolution images were captured using a Siemens 3T Trio scanner with a Magnetization Prepared Rapid Acquisition Gradient-echo sequence: inversion time of 900 ms, time of repetition/time of echo of 1900/2.52 ms, field of vision of 256 × 256 mm, flip angle of 9°, voxel size of 1 × 1 × 1 mm^3^ and 176 slices at a thickness of 1 mm. Individuals were asked to remain still to achieve optimal image quality.

### Voxel-based morphometry analysis

Images from structural MRI were analyzed using the Voxel-Based Morphometry (VBM) - DARTEL toolbox, implemented in SPM12 (v6906; Wellcome Trust Centre for Neuroimaging) through MATLAB 2015a (MathWorks Inc., Natick, Massachusetts, USA). Once verified that there were no artifacts or gross anatomical abnormalities, the images were adjusted manually to the anterior commissure orientation, with the purpose of improving registration. Subsequently, these images were segmented into white matter, grey matter, and cerebrospinal fluid with a novel segmentation algorithm, aimed to increase robustness and accuracy. Later, the images were resampled to 1.5 mm isotropic voxels, followed by registration through the DARTEL template-creation toolbox to enhance inter-subject alignment. After this, the images had to be standardized according to the Montreal Neurological Institute (MNI) standard space, then modulated by a Jacobian determinant derived from the degree of spatial normalization. Ultimately, the modulated images were smoothed with an isotropic Gaussian kernel with a Full-Width at Half-Maximum of 8 mm, reducing the artifacts and signal noise caused by image normalization and head motion. The grey matter regions present in this smoothed image were used to represent the grey matter volume (GMV). Subsequently, the resultant images were z-scored for further analyses.

It is worth mentioning that the most widely used Full-Width of isotropic Gaussian kernel is generally between 8 and 12 mm in previous studies. As Shen et al. concluded in their study, VBM analysis generally benefits from smaller kernels and different kernels perform best for different group sizes with a tendency of smaller kernels for larger groups. In their study, they found that in small sample size study, 8-10 mm kernel achieved the highest atrophy detection accuracy, while 6 mm kernel achieved the highest atrophy detection accuracy in dataset with sample size over 50. In our current study, considering the relatively small sample size, we therefore chose an isotropic Gaussian kernel with a Full-Width at Half-Maximum of 8 mm [24].

### Statistical analyses

**Fig. 1:**
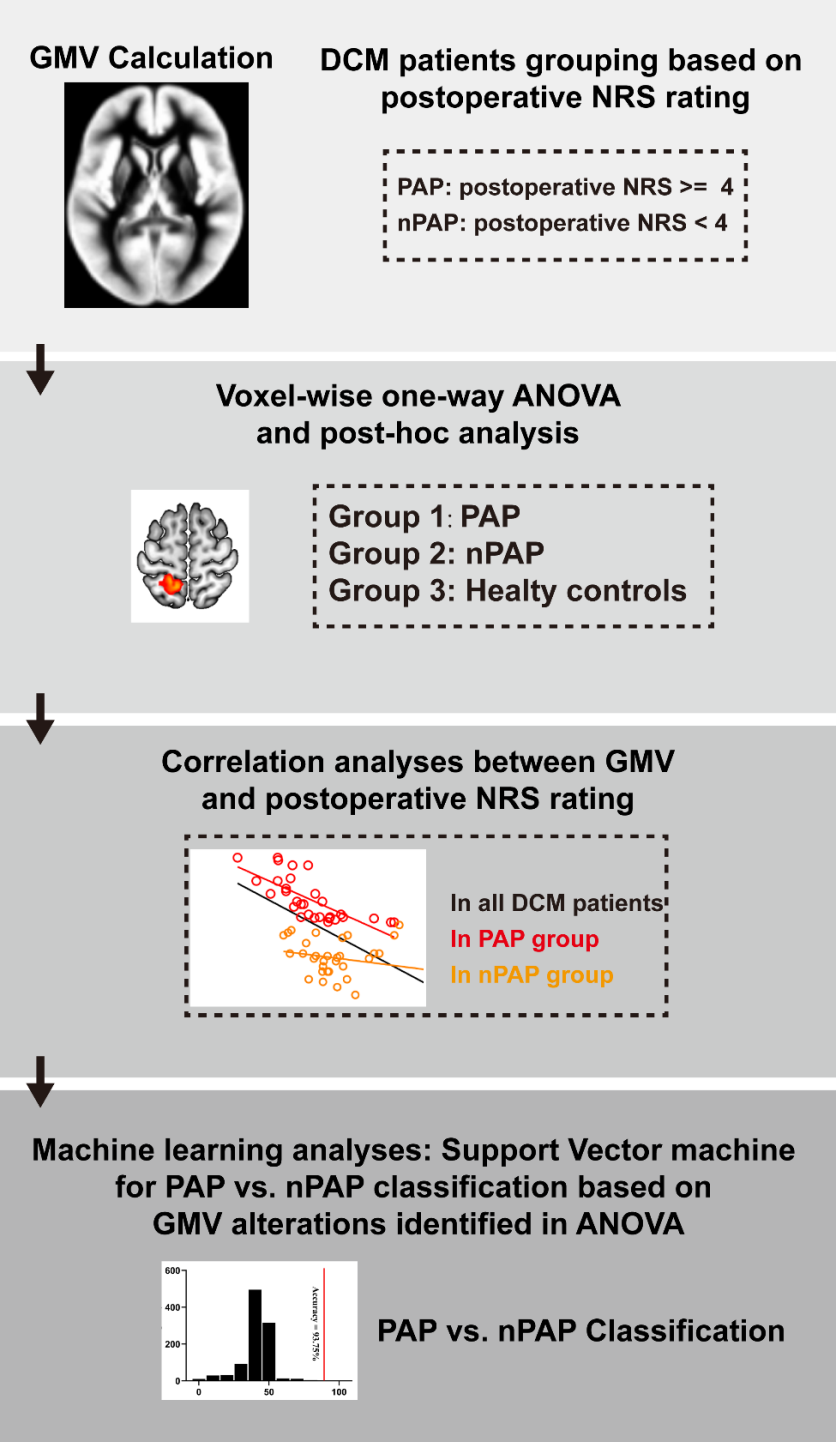
The analysis pipeline for our current study. In our current study, we calculated the grey matter volume (GMV) in all participants (both degenerative cervical myelopathy patients and healthy controls). We grouped the DCM patients into two subgroups based on postoperative numerical rating scale (NRS). Voxel-wise one-way ANOVA was performed, and post hoc analyses were performed. The mean GMV values within the resultant clusters were extracted and correlated with the clinical measurements in all patients, in PAP and nPAP. Finally, machine learning analyses were performed via support vector machine (SVM) for PAP vs. nPAP classification based on GMV alterations identified by ANOVA as features

The analysis pipeline of our current study could be found in Fig. 1. Voxel-wise one-way ANOVA was employed to contrast the GMV difference in a gray matter mask among PAP, nPAP group and healthy controls (HCs) with whole brain volume, age, gender, education years as covariates (P < 0.05, with false discovery rate correction using SPM12, http://www.fil.ion.ucl.ac.uk/spm). These demographic data (i.e., age, gender, education years) was also regressed out in other statistical analyses. The clusters surviving ANOVA were entered into Tukey Kramer test for post-hoc analyses to disclose the pairwise GMV differences among the three groups. To examine the correlations between brain function measures and clinical measures, Pearson correlation coefficients were computed separately for each patient group in regions of the brain that showed significant group differences. In addition, to test whether GMV could serve as a prognostic indicator for DCM patients to predict the occurrence of PAP following decompression surgery. Multi-variate pattern analysis (MVPA) was employed by Support Vector Machine (SVM) of MVPANI toolbox [25] using GMV as features extracted from brain regions identified in ANOVA.The leave-one-out cross-validation procedure (LOOCV) was performed to evaluate the accuracy of the classification. In LOOCV: First, a data-point in the available dataset was set-aside. Features were used to train a support vector machine model within the rest of the dataset and then the set-aside test data-point was used to test the model, therefore producing a predicted label for the test data point. This process was repeated until each data point was used once as the test data point. After that, an accuracy for this classification, which was determined as the proportion of accurate predictions out of total predictions were made, was employed to measure the effectiveness of the SVM model. To obtain the P-value, the labels of the subjects in the training dataset were randomly shuffled for 1000 permutation tests and a null distribution was generated from the associated feature set. To be specific, the P-value was determined by dividing the number of permutations that outmatched or were equal to the actual classification accuracy by the total number of permutations (1000). When no permutation yielded the desired accuracy, the p-value was considered to be less than 0.001. The detailed information for permutation procedure were as following: we used a permutation test method as following: [1] the labels of the individuals were haphazardly shuffled and categorized into two groups. Subsequently, classification analyses were performed via SVM; [3] the classification accuracy obtained from step 2 was then calculated. These procedures were repeated 1000 times to obtain a null distribution and P-value which was calculated as a fraction of the permutations that exceeded or equaled the real discrepancy, out of all 1000 permutations. If none achieved the precise divergence among the 1000 permutations, the p-value was taken to be less than 0.001.

## Results

### Demographic data

All participant demographics and clinical assessments are outlined in Table 1. In terms of age, sex, or education years, there were no significant intergroup differences (p > 0.05). No significant intergroup differences for PASS, FoP, PCS, PVAQ questionnaires. No significant difference in JOA was observed between PAP and nPAP group (T = 0.81, P = 0.42).

**Table 1:** Demographic data of the two groups

	DCM (n = 62)	HC (n = 42)	P-value
Age (years)	57.2 ± 8.17	57.1 ± 8.25	0.95
Sex (F/M)	31/31	21/21	1
Education (years)	12.9 ± 3.17	13.1 ± 2.53	0.73
JOA	11.2 ± 2.43	N/A	N/A
Diseases’ duration (months)	21.3 ± 15.14	N/A	N/A
Preoperative NRS	2.43 ± 2.46	N/A	N/A
Postoperative NRS	3.78 ± 2.56	N/A	N/A
FoP	90.1 ± 13.25	88.71 ± 14.32	0.61
PVAQ	34.12 ± 13.15	32.7 ± 12.41	0.58
PCS	12.78 ± 10.12	11.23 ± 9.81	0.43
PASS	37.42 ± 17.32	35.18 ± 15.23	0.49

DCM: degenerative cervical myelopathy; HC: healthy controls; NRS: numerical rating scale; JOA: Japanese Orthopaedic Association; FoP, fear of pain questionnaire; PVAQ, pain vigilance and awareness questionnaire; PCS, pain catastrophizing scale; PASS, pain anxiety symptom scale

### One-way ANOVA and post-hoc results

**Fig. 2:**
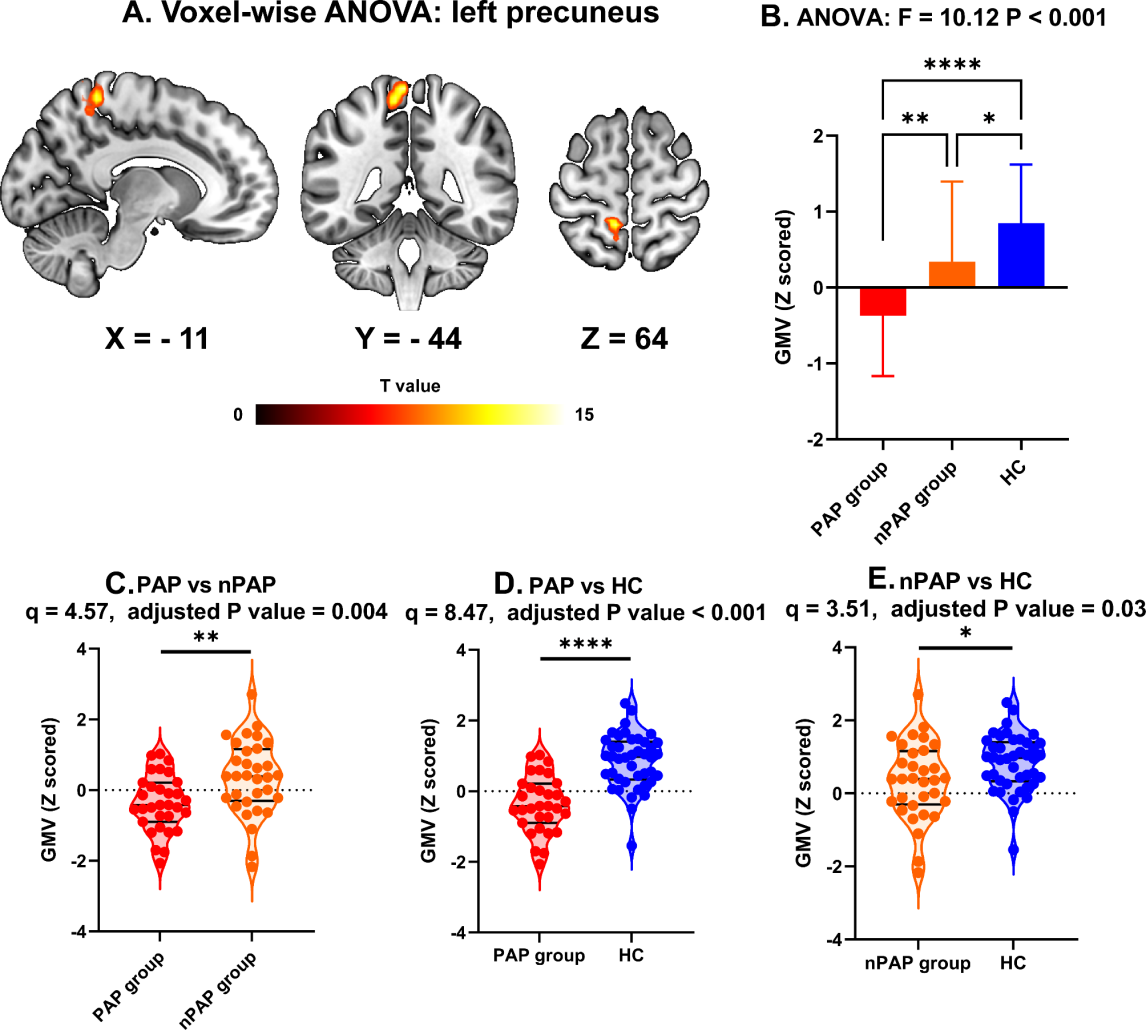
The brain regions that showed significant between-group differences in grey matter volume (GMV) among healthy controls (HCs), degenerative cervical myelopathy (DCM) patients with preoperative axial pain (PAP), and nPAP group

One-way ANOVA and post-hoc analyses were performed to reveal the GMV difference among PAP, nPAP, and HCs. There was an observable group effect in the left precuneus (F = 12.55) (Table 2; Fig. 2, panel A and B). The GMV of the clusters surviving ANOVA were entered into pairwise two-sample t tests in SPSS (IBM, version 23.0.0). After FDR correction, the GMV values of PAP and nPAP were significantly lower than that of HCs (Q = 8.34, adjusted P < 0.0001, Fig. 2, panel D; Q = 3.51, adjusted P = 0.03, Fig. 2, panel E), while a significant lower GMV were observed in PAP than nPAP (Q = 4.57, adjusted P = 0.0046, Fig. 2, panel C).

**Table 2:** The detailed information for GMV differences among DCM patients with preoperative axial pain, DCM patients without preoperative axial pain and healthy controls

Brain region	Brodmann area	MNI coordinates			Peak intensity	Voxel size
Left precuneus	BA 5	-13	-45	60	12.55	260

DCM: degenerative cervical myelopathy

### Correlation analyses

**Fig. 3:**
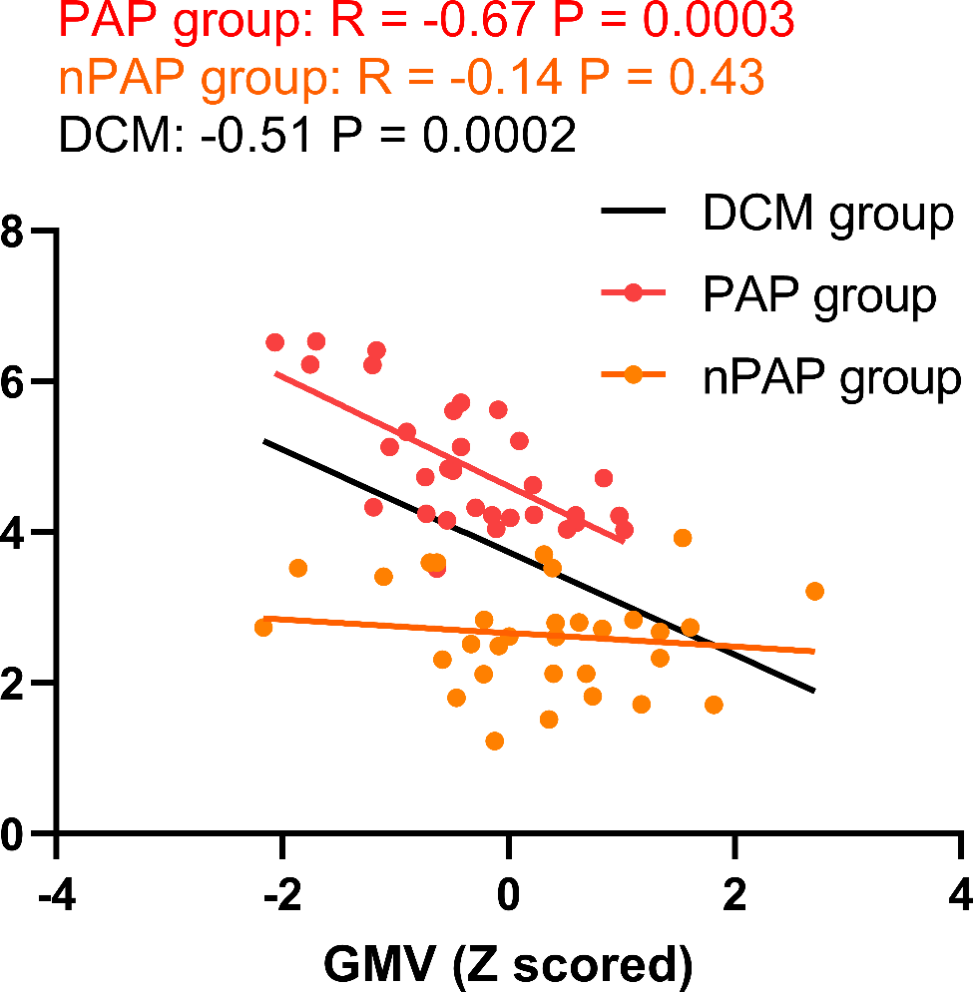
The association between postoperative numerical rating scale (NRS) ratings and grey matter volume (GMV) alterations in degenerative cervical myelopathy (DCM) patients with preoperative axial pain (PAP), without PAP, and in all DCM patients

To further reveal the association between GMV alterations and clinical assessments, Pearson correlation analyses were performed in our current study. A significant inverse relationship was noted between precuneus and postoperative NRS in DCM patients (R = -0.51, P = 0.0002, Fig. 3, Black line); and the GMV was also negatively correlated with postoperative NRS in PAP group (R = -0.67, P = 0.0003, Fig. 3, Red dots and line). There was no significant association between precuneus GMV and postoperative NRS in nPAP group (R = -0.14, P = 0.43, Fig. 3, Orange dots and line). No significant association was observed between precuneus GMV and final FoP score in our current study (Table 3). Further, no significant association was observed between preoperative pain intensity and brain alterations (All P > 0.1).

**Table 3:** Correlation between precuneus GMV and total FoP (fear of pain) score in DCM patients, preoperative axial pain (PAP), no preoperative axial pain (nPAP) and healthy controls

Precuneus GMV	R value	P value
In all DCM patients	-0.23	0.06
PAP	-0.28	0.12
nPAP	-0.08	0.65
Healthy controls	0.07	0.68

DCM: degenerative cervical myelopathy

### PAP vs. nPAP: SVM results

**Fig. 4:**
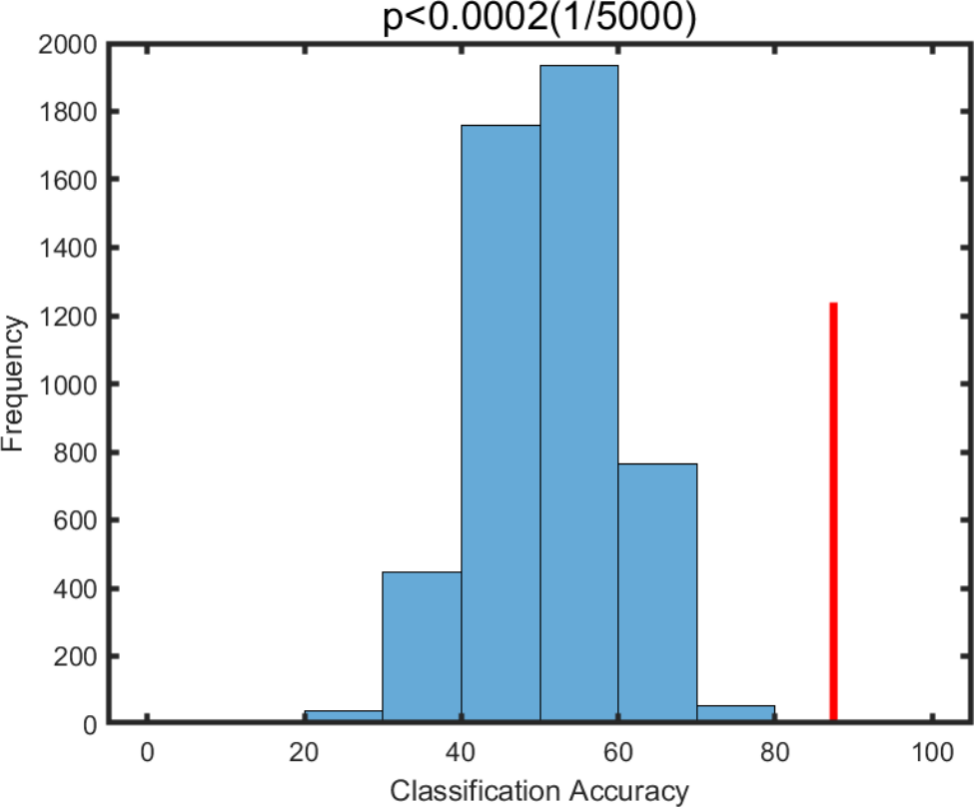
Machine learning analyses via support vector machine (SVM) for PAP vs. nPAP classification based on GMV alterations identified by ANOVA as features. True classification accuracy is 88.17%. The null distribution for permutations were illustrated and the classification accuracy were illustrated by red line. PAP: preoperative axial pain; GMV: grey matter volume

In machine learning analyses, the GMV values within precuneus identified in ANOVA were extracted as features for classifying PAP patients and nPAP patients. A significant classification accuracy was observed for this classification (Correct rate = 93.75%, P < 0.001, Fig. 4), and no accuracies obtained from permutations exceeded this classification accuracy.

## Discussion

The objective of the research was to investigate the brain structural connections associated with postoperative axial pain in DCM patients after postoperative decompression surgery. Three main findings were observed: [1] DCM patients showed a significant lower GMV in precuneus preoperatively in comparation to HCs. DCM patients with PAP also exhibited a lower GMV tin precuneus than those without; [2] In DCM patients with PAP, the precuneus GMV inversely associated with the postoperative pain intensity; [3] Additionally, successful classification between PAP and nPAP were observed via SVM based on precuneus GMV as features.

Precuneus, which has far-reaching anatomical and functional links to cortical and subcortical regions, is involved in higher order cognitive functions associated with pain such as the abilities to visualize, integrate multisensory information, retrieve episodic memory, and self-process information [26–29]. Particularly, given the widely acknowledged part of the precuneus in recording critical, self-relevant data [30–33]. It has been considered that precuneus, which is not present for the pain matrix, might associated with pain-related salient, self-relevant information rather than represent pain in the brain directly [13, 34, 35]. This assumption has been supported by recent research. Zhang et al. indicate that disparities in thermal pain sensitivity are attached to the precuneus of the DMN rather than the morphometry differences of the pain matrix [15]. This is evidence here to support the notion that precuneus is a system for detecting salience information rather than a part of nociceptive-specific system that is defined within the so-called pain matrix. In their study, the thermal pain threshold was positively correlated with the GMV value within precuneus, indicating that precuneus GMV negatively correlated with the individual variability to pain sensitivity.

In current study, we observed a significant lower GMV in patients with DCM, indicating that DCM patients might be more sensitive to pain. These results were not surprising and in line with previous study that the majority of the patients suffered from persistent pain which further modified the central nervous system (e.g., central sensitization), and accountable for variations in pain sensitivity [36–39]. Further, we also observed significant lower precuneus GMV in patients with PAP than those without, suggesting pain hypersensitivity in PAP patients. Despite no direct evidence were obtained in our current study for such assumption, previous studies have shown a reduced pressure pain threshold and temporal summation in patients with PAP comparison to those without PAP [9]. Their results suggested that a lack of endogenous pain regulation preoperatively may be linked to postoperative axial pain. Taken together, morphometry changes within precuneus might reflect preoperative pain central sensitization which associated with abnormal salience processing formed following preoperative chronic pain in DCM patients.

Further, to investigate utility of precuneus GMV pattern for classifying PAP and nPAP, SVM analyses were performed and successful classification was observed. In clinical practice, posterior cervical decompression surgery is a commonly used surgical approach [6], and the growing occurrence of PAP can seriously impact patients’ quality of life [40]. To date, the causes of PAP is still debate, and there is yet to be an accurate, objective way to predict PAP in those with DCM [7]. By identifying individuals with PAP, clinicians can develop new perioperative strategies to minimize or eliminate this complication associated with hypersensitivity in the cases. Researches have reported that the use of analgesics preoperatively may be effective to lessen pain intensity postoperatively in multiple orthopedic surgeries. This kind of perioperative preparation can decrease central sensitization, which could lead to a reduction in pain after major trauma [41, 42]. Our current finding provided new insights for developing novel imaging markers for monitoring the pain sensitization in DCM patients.

### Limitation

The primary limitation of the research is that all of the patients had already undergone drug treatment. This could potentially influence our findings to some degree. As such, it would be beneficial to do additional researches including DCM patients who takes no drugs or who have had a break in their medication regimen in order to confirm our results. Further, no postoperative images were acquired due to the possible heating and loosening of surgical implants, the postoperative data will be collected in the future when it’s safe.

## Conclusion

In conclusion, our results appear to show that precuneus GMV could be correlated with PAP in DCM, and could potentially be used to forecast PAP in those with DCM.

## Data Availability

The datasets used and/or analysed during the current study are available from the corresponding author on reasonable request.

## References

[1] Badhiwala JH, Ahuja CS, Akbar MA, Witiw CD, Nassiri F, Furlan JC (2020). Degenerative cervical myelopathy - update and future directions. Nat reviews Neurol.

[2] Iyer A, Azad TD, Tharin S (2016). Cervical spondylotic myelopathy. Clin spine Surg.

[3] Akter F, Kotter M (2018). Pathobiology of degenerative cervical myelopathy. Neurosurg Clin North Am.

[4] Davies BM, Mowforth OD, Smith EK, Kotter MR (2018). Degenerative cervical myelopathy. BMJ (Clinical research ed).

[5] Toledano M, Bartleson JD (2013). Cervical spondylotic myelopathy. Neurol Clin.

[6] Weinberg DS, Rhee JM (2020). Cervical laminoplasty: indication, technique, complications. J spine Surg (Hong Kong).

[7] Kimura A, Shiraishi Y, Inoue H, Endo T, Takeshita K (2018). Predictors of Persistent Axial Neck Pain after Cervical Laminoplasty. Spine.

[8] Wang SJ, Jiang SD, Jiang LS, Dai LY (2011). Axial pain after posterior cervical spine surgery: a systematic review. European spine journal: official publication of the european spine Society, the european spinal deformity Society, and the european section of the cervical. Spine Res Soc.

[9] Chen K, Yu J, Nie C, Zhu Y, Jiang J, Lei W (2022). Preoperative dynamic quantitative sensory testing in remote pain-free areas is associated with axial pain after posterior cervical spinal surgeries. BMC Musculoskelet Disord.

[10] Zhao R, Su Q, Song Y, Yang Q, Wang S, Zhang J (2022). Brain-activation-based individual identification reveals individually unique activation patterns elicited by pain and touch. NeuroImage.

[11] Chen J (2009). Toward the brain matrix of pain. Neurosci Bull.

[12] Iannetti GD, Mouraux A (2010). From the neuromatrix to the pain matrix (and back). Exp Brain Res.

[13] Legrain V, Iannetti GD, Plaghki L, Mouraux A (2011). The pain matrix reloaded: a salience detection system for the body. Prog Neurobiol.

[14] Jones SA, Morales AM, Holley AL, Wilson AC, Nagel BJ (2020). Default mode network connectivity is related to pain frequency and intensity in adolescents. NeuroImage Clin.

[15] Zhang X, Chen Q, Su Y, Meng J, Qiu J, Zheng W (2020). Pain in the default mode network: a voxel-based morphometry study on thermal pain sensitivity. NeuroReport.

[16] Khan SA, Keaser ML, Meiller TF, Seminowicz DA (2014). Altered structure and function in the hippocampus and medial prefrontal cortex in patients with burning mouth syndrome. Pain.

[17] Lin C, Lee SH, Weng HH (2016). Gray Matter Atrophy within the default Mode Network of Fibromyalgia: a Meta-analysis of Voxel-Based Morphometry Studies. Biomed Res Int.

[18] Liu J, Mu J, Liu Q, Dun W, Zhang M, Tian J (2017). Brain structural properties predict psychologically mediated hypoalgesia in an 8-week sham acupuncture treatment for migraine. Hum Brain Mapp.

[19] Shi H, Yuan C, Dai Z, Ma H, Sheng L (2016). Gray matter abnormalities associated with fibromyalgia: a meta-analysis of voxel-based morphometric studies. Semin Arthritis Rheum.

[20] Zhou XY, Xu XM, Wang F, Wu SY, Yang YL, Li M (2017). Validations and psychological properties of a simplified chinese version of pain anxiety symptoms scale (SC-PASS). Medicine.

[21] Yang Z, Meng J, Jackson T, Chen H (2013). Reliability and validity of the chinese version of fear of pain questionnaire - iii. Chin J Clin Psychol.

[22] Shen B, Wu B, Abdullah TB, Zhan G, Lian Q, Vania Apkarian A (2018). Translation and validation of simplified chinese version of the Pain Catastrophizing Scale in chronic pain patients: education may matter. Mol Pain.

[23] Wong WS, McCracken LM, Fielding R (2011). Factorial validity and reliability of the chinese version of the Pain Vigilance and Awareness Questionnaire (ChPVAQ) in a sample of chinese patients with chronic pain. Pain Med (Malden Mass).

[24] Shen S, Sterr A (2013). Is DARTEL-based voxel-based morphometry affected by width of smoothing kernel and group size? A study using simulated atrophy. J Magn Reson imaging: JMRI.

[25] Peng Y, Zhang X, Li Y, Su Q, Wang S, Liu F (2020). MVPANI: a Toolkit with friendly graphical user interface for Multivariate Pattern Analysis of Neuroimaging Data. Front Neurosci.

[26] Cavanna AE (2007). The precuneus and consciousness. CNS Spectr.

[27] Cavanna AE, Trimble MR (2006). The precuneus: a review of its functional anatomy and behavioural correlates. Brain.

[28] Cunningham SI, Tomasi D, Volkow ND (2017). Structural and functional connectivity of the precuneus and thalamus to the default mode network. Hum Brain Mapp.

[29] Tanglay O, Young IM, Dadario NB, Briggs RG, Fonseka RD, Dhanaraj V (2022). Anatomy and white-matter connections of the precuneus. Brain imaging and behavior.

[30] Del Casale A, Ferracuti S, Rapinesi C, Serata D, Caltagirone SS, Savoja V (2015). Pain perception and hypnosis: findings from recent functional neuroimaging studies. Int J Clin Exp Hypn.

[31] Gallace A, Bellan V (2018). The parietal cortex and pain perception: a body protection system. Handb Clin Neurol.

[32] Garcia-Larrea L, Mauguière F (2018). Pain syndromes and the parietal lobe. Handb Clin Neurol.

[33] Okada T, Kato D, Nomura Y, Obata N, Quan X, Morinaga A et al. Pain induces stable, active microcircuits in the somatosensory cortex that provide a therapeutic target. Sci Adv. 2021;7(12). 10.1126/sciadv.abd8261 PMC7978434 33741588

[34] Kim J, Mawla I, Kong J, Lee J, Gerber J, Ortiz A (2019). Somatotopically specific primary somatosensory connectivity to salience and default mode networks encodes clinical pain. Pain.

[35] Zeidan F, Lobanov OV, Kraft RA, Coghill RC (2015). Brain mechanisms supporting violated expectations of pain. Pain.

[36] Aoyagi K, Sharma NK (2021). Correlation between Central Sensitization and remote muscle performance in individuals with chronic low back Pain. J Manip Physiol Ther.

[37] LeBeau RT, Shaffer S, Earnshaw D. High-dose cervical mobilization to improve central sensitization for a patient with post-fusion neck pain. Physiother Theory Pract. 2021:1–8. 10.1080/09593985.2021.2015811 34895037

[38] Nunes A, Petersen K, Espanha M, Arendt-Nielsen L (2021). Sensitization in office workers with chronic neck pain in different pain conditions and intensities. Scandinavian J pain.

[39] Roldán-Jiménez C, Pérez-Cruzado D, Neblett R, Gatchel R, Cuesta-Vargas A. Central Sensitization in Chronic Musculoskeletal Pain Disorders in different populations: a cross-sectional study. Pain medicine (Malden, Mass). 2020;21(11):2958–63. 10.1093/pm/pnaa069 32232473

[40] Sun LQ, Li M, Li YM (2016). Predictors for Surgical Outcome of Laminoplasty for Cervical Spondylotic Myelopathy. World Neurosurg.

[41] Esparza-Villalpando V, Pozos-Guillén A, Masuoka-Ito D, Gaitán-Fonseca C, Chavarría-Bolaños D (2018). Analgesic efficacy of preoperative dexketoprofen trometamol: a systematic review and meta-analysis. Drug Dev Res.

[42] Carley ME, Chaparro LE, Choinière M, Kehlet H, Moore RA, Van Den Kerkhof E (2021). Pharmacotherapy for the Prevention of Chronic Pain after surgery in adults: an updated systematic review and Meta-analysis. Anesthesiology.

